# Effect of Exogenous Melatonin Administration in Critically Ill Patients on Delirium and Sleep: A Randomized Controlled Trial

**DOI:** 10.1155/2020/3951828

**Published:** 2020-09-23

**Authors:** Judith Bellapart, Vinesh Appadurai, Melissa Lassig-Smith, Janine Stuart, Christopher Zappala, Rob Boots

**Affiliations:** ^1^Department of Intensive Care Medicine, Royal Brisbane and Women's Hospital, Brisbane, Australia; ^2^Burns Trauma and Critical Care Research Centre, The University of Queensland, Brisbane, Australia; ^3^Department of Thoracic Medicine, Royal Brisbane and Women's Hospital, Brisbane, Australia

## Abstract

**Introduction:**

Sleep deprivation is a contributor for delirium in intensive care. Melatonin has been proposed as a pharmacological strategy to improve sleep, but studies have shown that the increase in plasma levels of melatonin do not correlate to a beneficial clinical effect; in addition, melatonin's short half-life may be a major limitation to achieving therapeutic levels. This study applies a previously published novel regimen of melatonin with proven sustained levels of melatonin during a 12 h period. In this study, the aim is to determine if such melatonin dosing positively influences on the sleep architecture and the incidence of delirium in intensive care.

**Methods:**

Single center, randomized control trial with consecutive recruitment over 5 years. Medical and surgical patients were in a recovery phase, all weaning from mechanical ventilation. Randomized allocation to placebo or enteral melatonin, using a previously described regimen (loading dose of 3 mg at 21 h, followed by 0.5 mg hourly maintenance dose until 03am through a nasogastric tube). Sleep recordings were performed using polysomnogram at baseline (prior to intervention) and the third night on melatonin (postintervention recording). Delirium was assessed using the Richmond Agitation and the Confusion Assessment Method Scales. Environmental light and noise levels were recorded using a luxmeter and sound meter.

**Results:**

80 patients were screened, but 33 were recruited. Sleep studies showed no statistical differences on arousal index or length of sleep. Baseline delirium scores showed no difference between groups when compared to postintervention scores. RASS scores were 1 in both groups at baseline, compared to zero (drug group) and 0.5 (placebo group) posttreatment. CAM scores were zero (drug group) and 1 (placebo group) at baseline, compared to zero (in both groups) postintervention.

**Conclusion:**

High levels of plasma melatonin during the overnight period of intensive care cohort patients did not improve sleep nor decreased the prevalence of delirium. This trial is registered with Anzctr.org.au/ACTRN12620000661976.aspx.

## 1. Introduction

The presence of a disrupted circadian rhythm within critically ill patients is one of the proposed pathways for the development of delirium in intensive care units (ICU). A constellation of factors such as loss of environmental zeitgebers, constant exposure to light or noise, and fragmented sleep architecture are concomitant contributors for ICU delirium [1]. The pathophysiological pathway seen in these scenarios comprises an altered secretion of endogenous melatonin, normally synthesized by the pineal gland and correlated to the duration of darkness and indirectly with its neuronal and sympathetic inputs [1]. In these situations, exogenous melatonin has been used as a pharmacologic strategy to improve sleep in the ICU [2–5]. Despite several studies investigating its clinical efficacy, the precise role of melatonin remains unclear. Authors have shown that a loss of circadian rhythm correlates with a reduction in the levels of plasma melatonin [6–15] and a reduced total serum concentration of endogenous melatonin in critically ill patients [9]. However, two studies have shown that supplementation of exogenous melatonin leads to a supraphysiological plasma concentration of melatonin, supporting its excellent bioavailability even during critical illness, although clinical translation is still lacking [16, 17].

Despite the extensive knowledge on the pharmacokinetics of melatonin [13, 18–23], to date, there are no studies showing a direct correlation between plasma levels of melatonin, quality of sleep, and reduction in the incidence of delirium [1]. Furthermore, the evidence supporting the use of melatonin in the ICU is confounded by the heterogeneity of the published studies' methodologies and the variability in their pharmacological regimens. Whilst, preserved oral bioavailability of melatonin and reduced plasma clearance in critically ill patients has been demonstrated [16] using an enteral 3 mg dose. A previous publication [17] demonstrated that exogenous administration of melatonin to a mixed cohort of patients, following a novel administration regimen resembling that of the pineal secretion lead to supraphysiological concentration of plasma melatonin over twelve hours, demonstrated good oral bioavailability amongst critically ill patients. This study attempted to reproduce the physiological area under the curve of the pineal secretion of melatonin based on the hypothesis that such “physiological mimicking” could bypass the spurious effect of exogenous melatonin ensuring supraphysiological plasma levels of melatonin for an entire night, promoting that way a better sleep and consequently, a reduction on delirium.

In the Cochrane systematic review on the promotion of sleep with administration of melatonin, a total of 4 studies [3, 4, 24, 25] were selected. However, the authors found insufficient evidence to determine whether the administration of melatonin improves the quantity and quality of sleep in the ICU. Furthermore, two clinical trials' protocols have been published [26, 27]. The pro-MEDIC study [26] is a multicenter RCT aiming to enroll 850 ICU patients, with 4 mg melatonin administered at 21 h for 14 consecutive days. Their aim is the use of melatonin for the prophylaxis of delirium and the enhancement of sleep, measured by polysomnography. The second trial [27] is a feasibility randomized study aiming to enroll 69 ICU patients and using a three arm (2 mg versus 0.5 mg melatonin versus placebo) intervention over 14 days, with the aim to prevent ICU delirium.

Despite all the studies conducted to date, the reasons behind the lack of clinical translation are unclear. It is plausible to think that the brief effect of melatonin due to its short half-life when administered in a noisy background, such as the ICU, may become ineffective to promote useful sleep. For such reason, the authors in this study, utilized the pharmacological regimen of melatonin from a previous pilot study [17] to assess its impact on the quality of sleep and the presence of ICU delirium.

## 2. Materials and Methods

This study was approved by the Royal Brisbane and Women's Hospital, Human Research Ethics Committee (HREC/09.QRBW/262). Written informed consent was obtained from all subjects' legal surrogates, as the investigators considered that the patients would not be cognitively competent to comprehend a consent process or had the potential to have delirium.

### 2.1. Study Hypothesis

1. Melatonin significantly increases the length and quality of sleep in critically ill patients exposed to less than 40 dB of noise and 10 lux of light (described as optimal environmental conditions)
2. Melatonin significantly reduces the incidence of ICU delirium

### 2.2. Study Population

A sample of critically ill patients in the “recovery phase” of their disease was consecutively recruited to a double-blind placebo-controlled trial of 6 mg enteral melatonin versus placebo via a nasogastric feeding tube. “Recovery phase” was defined as the resolution of the acute pathological process for which the patient had been admitted to the ICU and the initiation of weaning from mechanical ventilation. In this period of recovery, patients were no longer requiring vasopressors, and their sedative regimens were reduced with the aim to keep them awake during the day-light hours. All patients were still receiving mechanical ventilation, but they had already initiated a phase of weaning, so patients were in a spontaneous breathing mode. Few of these patients had received a tracheostomy in anticipation to a prolonged intubation. The inclusion of these patients at this phase of their disease is justified by the fact that often delirium is clinically manifested when sedatives are reduced in preparation to weaning of mechanical ventilation, delaying extubation, and ICU discharge. The selection of the sedation regimen was of the primary intensivist's discretion, and this was adherent to the unit's local protocol; therefore, all patients had a comparable sedative strategy.

Inclusion criteria also considered:

- Patients expected to have a minimal length of 5 days of respiratory weaning
- Patients with a preserved enteral absorption (defined as aspirates lesser than 400 ml 4th hourly) or the absence of ileus
- Patients without a known history of sleep disorders

### 2.3. Exclusion Criteria

Factors interfering with the metabolism of melatonin or an enhanced adrenergic state, included

- Beta-blockers, vasopressors, corticosteroids, nonsteroidal drugs, naloxone, or preintensive care prescription of antipsychotics
- Advanced liver disease
- Burns prior to debridement and grafts
- Ongoing sepsis
- Neurocritical patients

### 2.4. Study Drug Randomization and Administration

Randomization was blinded to the clinicians, to the main investigator, and to the research coordinators. Randomization had been achieved by the use of sealed envelopes containing two categories only known by the study's pharmacist who was responsible of preparing the study drug. The study drug was administered as a solution by solubilizing the contents of 3 mg capsules (Melatonin, Life Extension, USA) with water and administered through the patient's nasogastric feeding tube. A similar regimen was followed for the placebo administration. Both enteral solutions had the same visual characteristics. Both preparations were deidentified and were supplied by the ICU pharmacist in premade syringes. An administration record with unblinded allocation of the study drug to each patient was kept by the pharmacist, and this record was blinded to the research coordinators and to the investigators.

The study drug (melatonin or placebo) dosing began with a loading of 3 mg of melatonin or equivalent in volume, administered at 9pm; every hour after the initial loading dose, a 0.5 mg melatonin dose or its equivalent in volume was administered until a last dose at 3am. The total dose per night was equivalent to 6 mg as represented in Table 1. Such schedule intended to reproduce the endogenous nocturnal release of melatonin, which has been described to initiate in the evening and plateau after midnight [1]. Hourly enteral administration of the study drug was prescribed on the patients' medical orders in Metavision™ (Tel Aviv, Israel), the Clinical Information System (CIS) utilized at the time when this trial was recruiting; this systematic prescription ensured the compliance of the study drug administration by bedside nurses.

### 2.5. Sleep Recording

Patients' sleep was recorded with a polysomnogram (PSG) from evening (9pm) to morning rounds (6am) at two time points on the study: prior to intervention (“Baseline PSG”) and after three nights on melatonin (“Postintervention PSG”).

All sleep studies were analyzed by a sleep physician blinded to the clinical condition of the patient and the randomization group. PSGs was assessed for the length of stage 1–stage 3 non-REM sleep and the REM phase in proportion to the “total sleeping time” (TST), sleep latency, and arousals.

**Figure 1:**
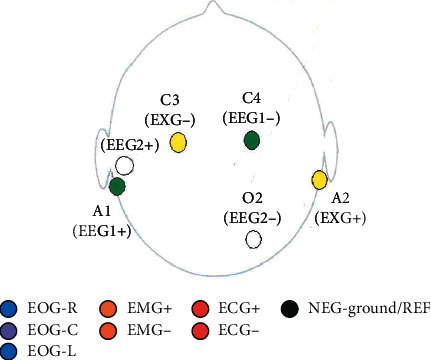
Polysomnogram montage. Basic six channels EEG montage for ICU-sleep studies with additional electroocular (EOG) leads in both eyes and electromyogram (EMG) at the trapezius muscles bilaterally.

Leads' montage was standardized. Leads' fixation to the skin utilized collodion, achieving an acceptable adherence to patients (Figure 1). Leads were placed at the evening shift, and the patient subsequently flagged to alert bedside nurses, optimize sleeping, and provide appropriate environmental conditions.

Sleep phases were described using the standard definition stages [28] and categorized as follows: stage 1, a light sleep phase where awakenings and hypnic jerks occur, muscle tone relaxes, and brain frequencies are alpha range (8–12 Hz). Stage 2, a 40–60% of total sleeping time in normal situations; a light but regenerating sleep phase with “sleep spindles”and “K-complexes” in theta range (4–8 Hz). Stage 3, a 5–15% total sleeping time where restorative sleep occurs and the glymphatic system activates, on delta range (1–4 Hz). REM stage, a phase of high frequencies' activity on a range of 15–30 Hz, where dreaming occurs, muscle tone is reduced, autonomic functions are activated, and long-term memory and learning are consolidated.

### 2.6. Delirium Recording

The categorization of delirium utilized the Richmond Agitation Sedation Scale (RASS) and Confusion Assessment Method (CAM), both validated and interchangeable [29]. Delirium was recorded as either “present” or “absent”. Assessments were performed by the principal investigator blinded to the intervention, always in the mornings and during the removal of the polysomnogram leads. RASS and CAM scales were implemented at two time points during the study: on the first sleep study (after the first night on study drug) and on the second and last polysomnogram recording (on the third night of study drug). Categorization of delirium took in consideration bedside observations from the treating team.

### 2.7. Environmental Variables

- Bedside light was maintained at the lowest levels possible for the night until 5am. Light levels were measured with a luxmeter (LM-81LX light meter, CE) at the horizontal level of the eyes prior to initiation of study. Patients could use welding glasses (Bolle safety shade 5) to ensure a maximum light exposure of 10 lux and to filter 460–470 nm wavelength light.
- Patients were offered industrial ear plugs, with the maximal background noise reduction of 32 dB (uvex X-fit, Uvex Arbeitsschutz, Germany). Noise measurements used an audiometer (IEC 651 Type II, CE) and maintained as low as possible by the bedside nurse. Bedside conversations were minimized, and an educational emphasis was implemented to ensure staff compliance.
- Recommendations focusing on the optimal timing for patients' hygiene were implemented regularly amongst nursing staff, inculcating the benefits of uninterrupted sleep.
- Patients' families were informed to minimize elective visits during sleeping hours.

### 2.8. Sample Size and Analysis

For this study, a significant reduction in relative risk was planned to determine a significant clinical signal. Using a reduction in delirium from 30% to 5% with a significance level of 5% and a power of 80%, 86 patients in total were meant to be enrolled. Data were analyzed on an intention to treat basis. The study was designed with the guidelines of the CONSORT statement to adhere to requirements for randomized controlled trials. Univariate data were described using medians with interquartile ranges (IQR) and 95% confidence intervals (95% CI) unless otherwise specified. Univariate analysis used chi-square, Fisher's exact, Wilcoxon rank sum, and Kruskal–Wallis tests as appropriate. The risk reduction was based on a clinically significant reduction in delirium and an achievable sample size for the initial study. Analysis was performed on patients completing the two studies, as this was the principle outcome in relation to the duration of treatment of the melatonin and the effects on the polysomnogram. No attempt was made to adjust the analysis due to the small sample remaining after attrition. Analysis used Stata 15.1 statistical software (College Station, Texas. United States of America).

## 3. Results

**Figure 2:**
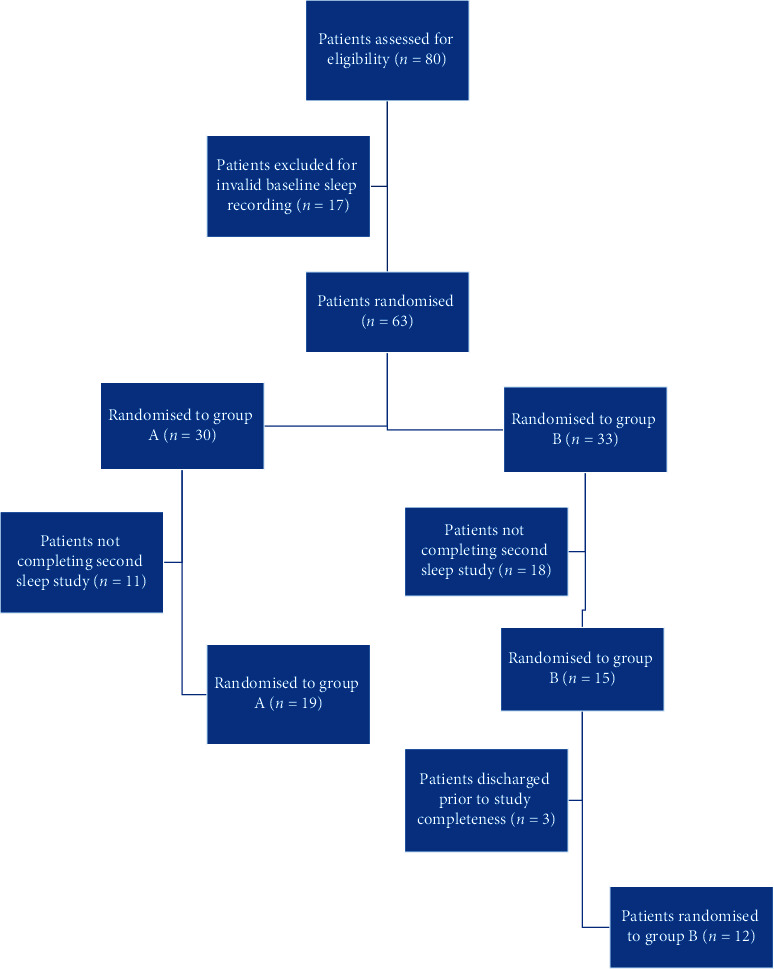
Consort diagram. Representation of the initially randomized but progressively missed patients.

A total of 80 critically ill patients were screened to this trial over a period of five years. A considerable number of patients had suboptimal baseline sleep recordings limiting their comparison with the post-intervention sleep study; therefore, these patients exited the trial. Only 63 patients were randomized to either melatonin or placebo. Two groups, group A (with 30 subjects) and group B (with 33 subjects), had a blinded allocation to either placebo or melatonin, only known to the ICU pharmacist. During the trial development, a large number of patients from both groups were extubated prior to the completion of the third night on the study drug and, consequently, prior to completing the second sleep recording. This patient attrition was the most significant limitation on this trial (Figure 2). The final number of patients having good quality sleep recordings, therefore, allowing their scoring, was distributed as group A with 19 patients and group B with 12 patients. These groups were comparable in age, gender, light, and noise exposure (Table 2). Patients' clinical severity was reflected by median APACHE II and III scores of 22 and 74, respectively, and a similar median ICU length of stay of 23 days.

**Figure 3:**
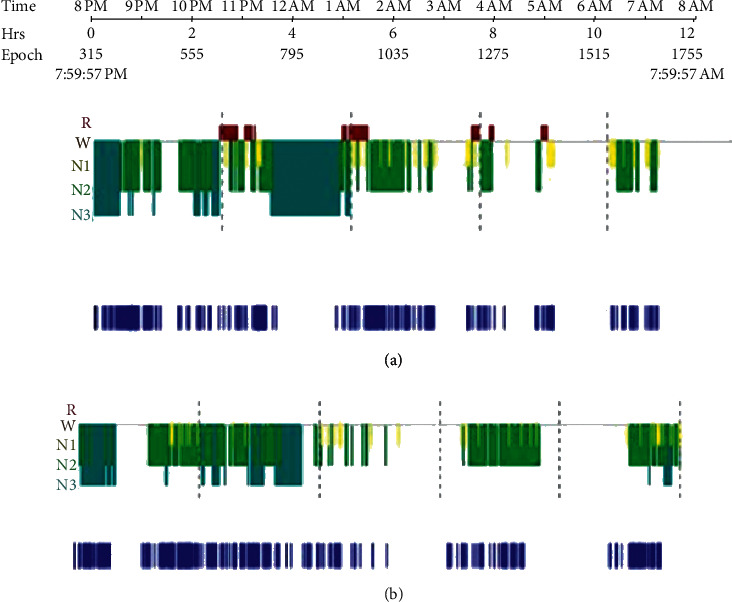
Control versus melatonin subject' hypnogram. R, REM stage; *N*1–*N*3, non-REM stages. Comparison between normal sleep stages in a healthy subject (hypnogram a) and one of the melatonin subjects (hypnogram b) showing absence of REM and non-REM sleep stages and presence of numerous arousals through all stages of sleep.

Baseline delirium and agitation scores showed no difference between groups when compared to postintervention scores. RASS scores were 1 in both groups at baseline assessments, compared to zero (in group A) and 0.5 (in group B) postintervention; with a change in RASS of zero between groups after intervention. CAM scores were zero (in group A) and 1 (in group B) at baseline, compared to zero (in both groups) postintervention; with a change in CAM of zero postintervention between groups, as shown in Table 3. The arousal index (number of arousals per hour of sleep) postintervention was not statistically different when compared to placebo or baseline (Table 4). The effect of the intervention was assessed for the proportion of each sleep stage in the polysomnogram and the “change” in the proportion of each sleep stage. There were no significant differences found on any sleep stages nor the period of wakefulness (Table 4). There was, however, poorly consolidated sleep with reduced stage 3 and REM sleep with frequent arousals (Figure 3).

## 4. Discussion

This trial aimed to find a reduction in ICU-related delirium, through the potential benefits of sleep by using a novel regime of melatonin in critically ill patients in a recovery phase of their disease.

The authors had hypothesized the advantage of administering melatonin in a novel regime, which by scheduling multiple administrations as opposed to a once only dose, plasma levels of melatonin could achieve a supraphysiological rise and be maintained over a 12 h period (from 9pm to 9am). With such administration regime, the investigators were attempting to simulate a more physiologic dosing pattern. This group of investigators had previously demonstrated [17] that such novel regime had very good bioavailability providing supraphysiological concentrations of melatonin (up to 10 folds above the placebo group) with the additive effect of multiple doses with high concentrations in plasma for 12 h overnight.

The main outcome measure in this trial was the prevalence of ICU-related delirium measured by RASS and CAM scales. In this trial, the investigators' hypothesis was that by improving sleep, patients would develop significantly less delirium. However, in this cohort of patients, melatonin had no effect on the reduction of postintervention delirium. It is plausible that regardless the promising pharmacokinetics of oral melatonin, the presence of very high light and noise levels at patients' bedside and ongoing sleep interruptions could mitigate the potential effects of melatonin. This phenomenon would emphasize the need of preserving sleep hygiene and environmental conditions to promote sleep in the critically ill. Little is known of the pharmacological interaction of common mixtures of sedative, anxiolytic, antipsychotic, and analgesic regimens commonly used in critically ill patients during ventilation weaning. It is unclear if the maintenance of such pharmacological mixtures could have influenced delirium and depression and the effectiveness of melatonin.

The second outcome measure of this study was the polysomnogram analysis of patients' sleep. First, no significant differences were seen amongst sleep stages and randomized groups. The additional administration of melatonin did not increase the length of deep sleep nor modify the sleep pattern when compared to the baseline and placebo group. The proportion of sleep stages were abnormal compared to normal sleep. There was a 0% REM phase in both groups with a high proportion of time spent awake. Intensive care units are noisy and bright environments where the delivery of very advance resuscitation strategies coexists with the need for proper sleep that allows recovery. It is well known that sleep deprivation has deleterious effects on ICU delirium, increased length of stay in ICU, and post-ICU stress disorder. Despite, international strategies focusing on designing intensive care units were the patient's bedside environment that can dynamically change to adequate to the patients' needs, current pharmacological armamentarium, and daily practices that fail to provide the conditions for proper sleep. In our trial, stage 2 was most common than stage 3, again, showing that lighter sleep predominates over deep restorative sleep. When combined stages 2 and 3, their overall proportion was underrepresented, as arousals and awake phases were most lengthy over the sleeping time.

Melatonin did not shorten the proportion of arousals. Arousals are detrimental to a proper sleep pattern because they reset the person back to a lighter stage in their sleep. Furthermore, when arousals are increased, they become awakening; therefore, the higher the arousal index, the tired even a healthy person will be. This has serious implications in ICU patients considering that their sleeping time is commonly fragmented and interrupted by healthcare staff due to elective activities. To date, this globally adopted practice is at the spotlight of research and may be a guide towards future ICU designs.

Our study had several limitations. First, protocol violation was identified during recruitment and data collection including despite eye pads, sunglasses, and ear plugs offered to patients; due to perception of isolation and discomfort, they became unpopular amongst nursing staff. In addition, high levels of sound and light were recorded at patients' bedside. Culture shift with regards to nursing activities (scheduled hygiene) and environmental quality were not achieved. There was a long recruitment period related to the number of patients exiting the trial due to extubation prior to the second PSG, and this was a significant contributor to the final attrition rate. The time point for the postintervention sleep recording was arbitrarily chosen by the investigators, and it was considered that three days and nights could suffice for a steady-state on the awake-sleep cycle; however, it is very likely that critically ill patients' circadian rhythm is impaired beyond ICU discharge, and thus, the lack of the beneficial effect of melatonin could have been also related to the timing upon which the outcome measure was assessed. It is unknown if discharging these patients from the ICU on melatonin could lead to a significant effect later through patients' recovery in the hospital wards. Due to the attrition rate, the final sample size for analysis was too small to demonstrate any effect of the intervention. This trial, the multifactorial contributors for the quality of sleep and the development of delirium in the ICU were not controlled. The pragmatical approach taken in the design of this trial may have introduced confounders, which need to be considered when interpreting its findings.

## 5. Conclusion

Enteral administration of melatonin in critically ill patients, using a novel regime that achieves supraphysiological levels in plasma, does not improve the length of deep sleep, does not increase the presence of the REM phase, nor minimizes the incidence of arousals. In addition, this regimen of enteral melatonin does not reduce the prevalence of ICU-related delirium.

## Figures and Tables

**Table 1:** Study drug administration regimen.

Real drug administration time schedule	Measurement/sample
21 h	First 3 mg dose of study drug
22 h	Administration of 0.5 mg study drug
23 h	Administration of 0.5 mg study drug
24 h	Administration of 0.5 mg study drug
01 h	Administration of 0.5 mg study drug
02 h	Administration of 0.5 mg study drug
03 h	Administration of 0.5 mg study drug

**Table 2:** Epidemiologic and environmental characteristics of the studied cohort.

Parameters	Group A	Group B	Total	*P*
	Melatonin	Placebo		
	*N* = 21	*N* = 12	*N* = 33	
	Median (IQR)	Median (IQR)	Median (IQR)	
Age (years)	55 (43–66)	57.5 (50–64)	55 (49–66)	0.50
APACHE II	22 (12–30)	24 (14–28)	22 (14–29)	0.81
APACHE III	74 (53–94)	78 (47–101)	74 (53–94)	0.90
ICU length of stay (days)	24 (16–36)	23 (16–35)	23 (16–36)	0.71
Study ICU enrollment day	8 (6–15)	10 (3–14)	10 (3–14)	0.62
Light intensity (lux)*∗N* = 14	50 (25–60)	50 (30–65)	50 (25–63)	0.69
Sound intensity (dB)*∗∗N* = 14	60 (50–62)	65 (65–70)	62 (50–65)	0.12

*∗*lux, luxes; *∗∗*dB, decibels.

**Table 3:** CAM and RASS scores distributed per randomized groups.

Parameter	Group A	Group B	*P*
	*N* = 21	*N* = 12	
	Melatonin	Placebo	
	Median (IQR)	Median (IQR)	
RASS			
Baseline	1 (0–1)	1 (0–1)	0.43
Posttreatment	0 (0–1)	0.5 (0–1)	0.12
Change from baseline to posttreatment	0 (−1–0)	0 (−1–0)	0.61
CAM			
Baseline	0 (0–1)	1 (0.5–1)	0.08
Post treatment	0 (0–0)	0 (0–1)	0.11
Change baseline to post treatment	0 (−1–0)	0 (−1–0)	0.61

RASS, Richmond Agitation Sedation Scale; CAM, Confusion Assessment Method.

**Table 4:** Sleep analysis in study groups.

	Group A	Group B	*P*
	*N* = 21	*N* = 12	
	Melatonin	Placebo	
	Median (IQR)	Median (IQR)	
Hrs studied from 7pm, baseline	10 (10–10)	10 (10–10)	0.80
Hrs studied from 7pm, drug	10 (10–10)	10 (10–10)	0.57
Arousal index			
Baseline	8.95 (6.6–20.3)	7.6 (6.6–20.3)	0.80
Posttreatment	12.9 (8.4–24.4)	15.7 (1.8–66)	0.81
Sleep stages	*N* = 14	*N* = 8	
Stage 1 (%)			
Baseline	0.26 (0.00–1.00)	0 (0.0–1.7)	0.37
Posttreatment	2.17 (0.75–3.25)	0.25 (0.0–2.03)	0.10
Change from baseline to posttreatment	0.89 (−0.33–2.42)	0.17 (−0.04–0.60)	0.39
Stage 2 (%)			
Baseline	24.67 (6.93–40.08)	2.92 (0.17–15.75)	0.02
Posttreatment	21.17 (15.57–43.15)	16.30 (6.35–31.25)	0.13
Change from baseline to posttreatment	−3.49 (−9.27–15.83)	9.35 (1.56–27.18)	0.25
Stage 3 (%)			
Baseline	11.67 (5.23–54.81)	7.00 (0.50–83.33)	0.94
Posttreatment	16.50 (1.92–29.33)	54.58 (11.05–59.52)	0.02
Change from baseline to posttreatment	17.54 (1.92–29.33)	46.03 (10.3–58.17)	0.08
REM (%)			
Baseline	0 (0–0.08)	0 (0–0)	0.32
Posttreatment	1.05 (0–4.42)	0 (0–0)	0.05
Change from baseline to posttreatment	0.13 (0–1.5)	0 (0–0)	0.35
Awake (%)			
Baseline	32.50 (13.07–58.5)	69.08 (13.86–96)	0.62
Posttreatment	45.69 (34.02–70.67)	21.29 (3.50–61.63)	0.22
Change baseline to posttreatment	8.34 (−13.17–15.42)	−11.56 (−47.10–3.93)	0.22

## Data Availability

The datasets supporting the conclusions of this article are available from the corresponding author upon reasonable request.
